# Complete genome sequence of a *bla*_OXA-1_*-*carrying *Escherichia coli* from Laguna Lake, Philippines

**DOI:** 10.1128/mra.00006-26

**Published:** 2026-04-08

**Authors:** Aryana Lee G. Bertuso, Michael Joseph M. Nagpala, Aira Charlien R. De Guzman, Kherson P. Gandola, Patrick Philip A. Salvador, Laurice Beatrice Raphaelle O. dela Peña, Windell L. Rivera

**Affiliations:** 1 Pathogen-Host-Environment Interactions Research Laboratory, Institute of Biology, College of Science, University of the Philippines Diliman 54727, Quezon City, Philippines; Fluxus Inc., Sunnyvale, California, USA

**Keywords:** *Escherichia coli*, *bla*_OXA-1_, Laguna Lake, antimicrobial resistance, complete genome

## Abstract

We report the complete genome sequence of *Escherichia coli* L16-131 (4,850,597 bp chromosome) with five circular plasmids, isolated from Laguna Lake, Philippines. The isolate harbored multiple antimicrobial resistance genes (ARGs); most were computationally predicted to be carried by p1_L16-131.

## ANNOUNCEMENT

The *bla*_OXA-1_ gene encodes a clinically significant β-lactamase that confers resistance to penicillins and cephalosporins, often found in Enterobacteriaceae resistomes, particularly on plasmids and integrons (1, 2). Investigating its occurrence in environmental settings, especially in freshwater ecosystems such as Laguna Lake, is crucial for assessing its potential role in the dissemination of antimicrobial resistance (AMR) (3). This study presents the complete genome of a *bla*_OXA-1_-carrying *Escherichia coli* from the Philippines, providing baseline genomic data that support AMR surveillance efforts and enhance understanding of resistance gene mobility in aquatic environments.

The *uidA*-positive *E. coli* isolate was obtained from Laguna Lake Station XVI (West Bay), Sta. Rosa, Laguna, Philippines (14.329336, 121.131993), following Laguna Lake Development Authority regulations and institutional requirements. Subsurface water samples were collected, membrane-filtered, and cultured on modified mTEC agar (BD Difco), followed by purification and DNA extraction by boil-lysis (4). The target *bla*_OXA-1_ gene was amplified using previously described primers with modified annealing at 62°C (5). A colony from Nutrient Agar was inoculated into Tryptic Soy Broth and incubated at 37°C for 24 hours. Afterward, cells from a 12-mL culture were washed with PBS, resuspended in 500 μL Zymo DNA/RNA Shield (Zymo Research), and were sent to Plasmidsaurus for hybrid sequencing. DNA was extracted using *Quick*-DNA Fungal/Bacterial Miniprep Kit (Zymo Research). Libraries were prepared using Rapid Barcoding Kit (SQK-RBK114.96; Oxford Nanopore Technologies) and Illumina DNA Prep Kit (20060059; Illumina). Long-read sequencing was performed on PromethION P24 (R10.4.1 flow cell) and basecalled with ont-dorado-for-promethion v7.4.12, while 150-bp paired-end short reads were generated on a NextSeq 2000.

Long reads were filtered using Chopper v0.10.0b (6). Reads were assembled with Autocycler v0.5.2 (7), with filtered reads subsampled into four subsets targeting a 5 Mbp genome using “autocycler subsample”. These subsets were assembled with Canu v2.3 (8), Flye v2.9.6-b1802 (9), Miniasm v0.3-r179 (10), and Raven v1.8.3 (11), while Plassembler v1.8.0 (12) was used to recover small plasmids. Autocycler then constructed a compacted de Bruijn graph (“autocycler compress”), clustered contigs by pairwise graph-path distances (“autocycler cluster”), removed low-confidence clusters, trimmed circular and hairpin overlaps (“autocycler trim”), resolved repeats (“autocycler resolve”), and merged the resulting consensus contigs into a final assembly (“autocycler combine”). Illumina reads were quality-checked using FastQC v0.12.1 (13) and trimmed with fastp v1.0.1 (14). The trimmed reads were then used to refine the long-read assembly using Polypolish v0.6.1 (15). The origins of chromosome and plasmids were determined using BAKTA (16), and circular replicons were rotated to these positions using the “rotate” command. Annotation was performed using the NCBI Prokaryotic Genome Annotation Pipeline v6.10 (17), and ARGs were cross-validated using ResFinder v4.7.2 (18). Default parameters were applied unless stated otherwise.

**Fig 1:**
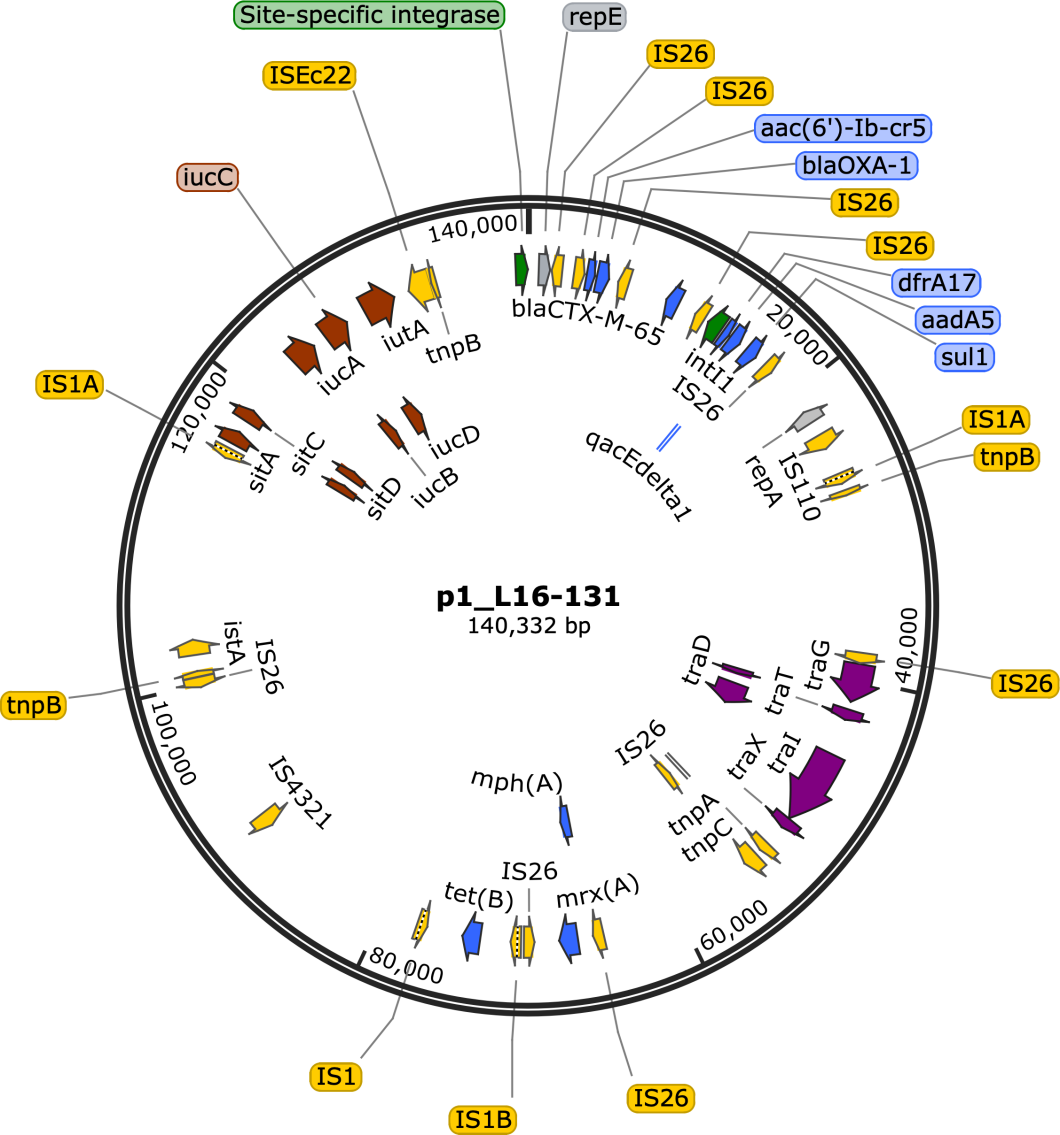
Genetic map of the 140,332 bp plasmid (p1_L16-131) of *E. coli* strain L16-131 from Laguna Lake Station XVI, Philippines. The map highlights antimicrobial resistance genes in blue, virulence genes in red, *tra* genes in purple, integrase in green, and transposase in yellow. Key antimicrobial resistance genes and their functions include: *aac(6′)-Ib-cr5,* fluoroquinolone-acetylating aminoglycoside 6′-N-acetyltransferase; *bla*_OXA-1_*,* oxacillin-hydrolyzing class D beta-lactamase; *bla*_CTX-M-65_*,* extended-spectrum class A beta-lactamase; *dfrA17,* trimethoprim-resistant dihydrofolate reductase; *aadA5,* ANT(3′′)-Ia family aminoglycoside nucleotidyltransferase; *qacEdelta1,* quaternary ammonium compound efflux SMR transporter; *sul1,* sulfonamide-resistant dihydropteroate synthase; *mph(A),* macrolide 2′-phosphotransferase; *mrx(A),* macrolide resistance MFS transporter; *tetB,* tetracycline efflux MFS transporter. Genes were computationally predicted and annotated using NCBI Prokaryotic Genome Annotation Pipeline v6.10, cross-validated with ResFinder v4.7.2, and plotted using SnapGene Version 8.0.3.

A total of 8.6 M short-read sequences (1.2 Gbp) and 81.9 K long-read sequences (726 Mbp; N50 = 15.8 kbp; mean quality = 20.8) were used to assemble the *E. coli* genome. The final assembly yielded a closed, circular 4,850,597 bp chromosome (50.93 %GC), and five circular plasmids (Table 1). The genome harbored multiple ARGs, most of which are on p1_L16-131 (Fig. 1).

**TABLE 1:** Genomic characteristics of *E. coli* strain L16-131 from Laguna Lake station XVI, Philippines

Parameters	Genomic features
Genome	Total Nanopore coverage: 146X
	Total Illumina coverage: 516X
Chromosome	Length: 4,850,597 bp
	Nanopore coverage: 138X
	Illumina coverage: 510X
	GenBank acc. no.: JBSJPC010000001.1
Plasmids	
p1_L16-131	Length: 140,332 bp
	Nanopore coverage: 75X
	Illumina coverage: 274X
	GenBank acc. no.: JBSJPC010000002.1
p2_L16-131	Length: 14,561 bp
	Nanopore coverage: 2,230X
	Illumina coverage: 2,169X
	GenBank acc. no.: JBSJPC010000003.1
p3_L16-131	Length: 3,134 bp
	Nanopore coverage: 3,023X
	Illumina coverage: 5,262X
	GenBank acc. no.: JBSJPC010000004.1
p4_L16-131	Length: 1,597 bp
	Nanopore coverage: 3,221X
	Illumina coverage: 9,280X
	GenBank acc. no.: JBSJPC010000005.1
p5_L16-131	Length: 1,551 bp
	Nanopore coverage: 3,473X
	llumina coverage: 7,275X
	GenBank acc. no.: JBSJPC010000006.1
Annotation	
CDS	4,807
rRNAs	22
tRNAs	87
ncRNAs	12
Antimicrobial resistance genes	
Chromosome	*ampC*
Plasmids	
p1_L16-131	*aac(6′)-Ib-cr5*, *bla*_OXA-1_, *bla*_CTX-M-65_*, dfrA17, aadA5, qacEdelta1, sul1, mph(A), mrx(A), tetB*

## Data Availability

The complete genome sequence has been deposited in GenBank under accession no. GCA_053668395.1 and in RefSeq under accession number GCF_053668395.1. The corresponding sequence records are JBSJPC010000001–JBSJPC010000006. The raw sequencing data have been deposited in the NCBI Sequence Read Archive under accession no. SRX31124910 (short reads) and SRX31124909 (long reads). The genome is associated with BioProject PRJNA1365059 and BioSample SAMN53269467.
